# Influence of sex on survival rates of HPV-positive oropharyngeal cancers

**DOI:** 10.3389/fonc.2022.917890

**Published:** 2022-08-10

**Authors:** Sally H. Preissner, Susanne Nahles, Saskia Preissner, Max Heiland, Steffen Koerdt

**Affiliations:** Department of Oral and Maxillofacial Surgery, Charité – Universitätsmedizin Berlin, Corporate Member of Freie Universität Berlin, Humboldt-Universität zu Berlin, and Berlin Institute of Health, Berlin, Germany

**Keywords:** head and neck cancer, oropharyngeal cancer, oral squamous cell carcinoma, sex, HPV - human papillomavirus

## Abstract

The role of human papillomavirus (HPV) status for the prognosis of oropharyngeal cancers (OPCs) is discussed controversially. Here, we present an analysis of 144,969 head and neck cancer cases (ICD-10 codes: C00–C13) with a sub-cohort of 62,775 tumor cases of the oropharynx (C01, C09, and C10). To this end, de-identified data from electronic health records of about 60 healthcare organizations from 30 different countries were used. Odds ratios, hazard ratios (HRs), and Kaplan–Meier analyses were used to compare outcomes between different cancer entities of neoplasms of the base of the tongue (C01), of tonsils (C09), and of the oropharynx (C10) of women and men with and without HPV infection. To avoid the bias from different age distributions, the cohorts were balanced using propensity score matching. The 5-year survival rate for HPV-positive patients is somewhat better than that for HPV-negative patients, but for age- and sex-balanced cohorts, there remains no significant advantage for HPV-positive patients [HR, 1.126 (0.897–1.413)]. Looking at the different entities and HPV status for age-matched male and female patients separately, HPV is a significantly positive prognostic factor for female patients in some entities, whereas for male patients, it is only a positive prognostic factor for malignant neoplasms of oropharynx (C10) [HR, 1.077 (0.602–1.926)].

## Introduction

Head and neck cancers, most of which are histologically squamous cell carcinomas, consist of cancers of the oral cavity, nasopharyngeal, hypopharynx, and oropharynx. Oropharyngeal cancers (OPCs) are one of the most common subtypes of head and neck cancers (1). Although the total incidence decreases, due to the decline in traditional risk factors such as nicotine or alcohol, the incidence of HPV-positive OPC has tripled in the United States between 1988 and 2004 and overhauled vaginal and cervical cancer incidence (2).

In the United States and Western Europe, 70%–80% of all OPCs are associated with HPV (3). OPCs include carcinomas of the tonsils, the base of the tongue, the posterior pharynx wall, and the soft palate (4). For healthy patients, the prevalence of an oral HPV infection is approximately 6.1%, including 1% HPV16, with a higher prevalence in men (10.1%) than in women (3.6%) (5).

Generally, patients are younger and healthier and have a significantly lower incidence of traditional risk factors such as smoking and alcohol abuse (6, 7).

Clearance of the virus was observed for 90% of the patients within 1 to 2 years (8). In the remaining 10%, the infections persist with a risk of malignant transformation (9). Because HPV is a sexually transmitted disease (STD), sexual behavior is considered as the major risk factor for HPV-positive OPC (10). The number of oral sexual intercourse partners is the strongest associated factor (10). Partners of patients with HPV-positive OPC do not seem to have a higher risk of a persisting HPV infection because they are able to eliminate the virus (11, 12). The fact of men being more likely to have an HPV-positive OPC is probably related to the number of copies of HPV in vaginal and cervical tissues being higher than on the penis (13).

### HPV infection and carcinogenesis

More than 200 genotypes of HPV have been identified, of which 15 are classified to have oncogenic potential (14). HPV has a tropism for squamous epithelium, and it is limited to the basal cells of the stratified epithelium and infects epithelial tissues through micro–abrasions and epithelial trauma after sexual transmission (15).

The diagnosis of HPV is challenging as most OPCs have small primary tumor sizes. Although it is extensive and, in many cases, involves the lymph node, the diagnosis is usually made in progressed stages due to swollen lymph nodes (16).

On the one hand, according to the prevailing view in literature, HPV–associated oropharyngeal carcinoma is linked with a more favorable prognosis when compared to HPV–negative disease (17). On the other hand, after comparing different studies, it is noticeable that HPV–positive OPC patients are younger at the point of diagnose and had less comorbidities (or regardless of comorbidities) than HPV–negative OPC patients (18).

As sex has been identified as an independent parameter in the onset of HPV–positive OPC, up to date, no studies about the influence of sex on long–term survival rates are available, this current study contains an analysis of HPV–positive OPC according to age and sex with respect to survival rates during 5–year follow–up.

## Methods

### Database and inclusion criteria

This work distributes a retrospective analysis of an international head and neck cancer cohort including 144,969 cases. Sub–cohorts were defined by HPV status and male or female cases. Patients with head and neck cancer were identified *via* the ICD–10 code (C00–C13), a sub–cohort for OPC was generated using ICD–10 codes C01, C09, and C10. In addition, cohorts of HPV–positive and HPV–negative patients were carefully defined on the basis of criteria, which can be found in the **Supplementary Material**. The data were gathered from electronic health records out of the TriNetX Real World database. The real–world evidence (RWE) has been provided by a global health research network with 40 healthcare organizations allocated in 30 countries, which represent a continually updated global health network of over 300 million patients.

### TriNetX

The TriNetX platform assures the quality of data to be controlled by processes and procedures triggered in response to questions about the data provided. TriNetX combines longitudinal clinical data and analytics to help in generating RWE. Datasets do not leave hospitals: queries are executed in a federated manner, and only aggregated results are visible on TriNetX.

### Statistical analysis

TriNetX analytics tools were used to obtain baseline characteristics, to balance cohorts with propensity score matching, and to analyze outcomes of interest in the final cohorts. The index event for each analysis was selected as the diagnosis of head and neck cancer (ICD 10: C00–C13) within the last 5 years excluding adenocarcinomas. Baseline characteristics, including demographics and diagnoses, were obtained. To compare the outcomes of different cancer entities for men and women with and without HPV infection, we applied hazard ratios (HRs) with 95% confidence intervals (CIs) and generated Kaplan–Meier curves. Propensity score matching was used to balance cohorts in address confounders that could bias analysis. Cohorts were balanced for age, alcohol, and nicotine dependence. Propensity scores matched cohorts 1:1 using a nearest neighbor greedy matching algorithm with a caliper of 0.25 times the standard deviation. This 1:1 matching was conducted to replicate randomized conditions as closely as possible. The primary outcome was defined as death.

## Results

**Figure 1:**
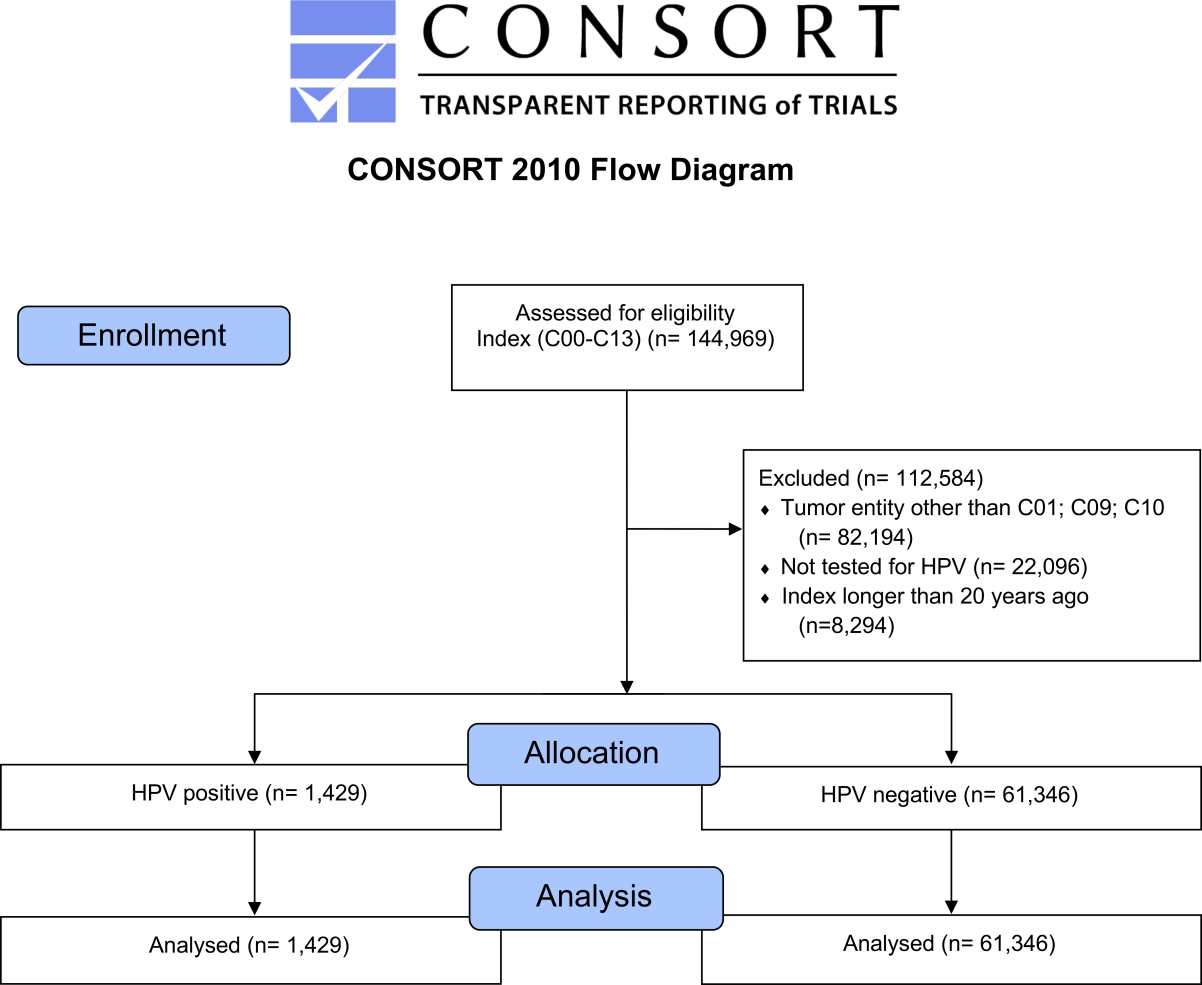
CONSORT flow diagram, major stages are indicated in the blue boxes. Overall, tumors of the tongue base had the highest frequency followed by mouth and tonsils (**Table 1**). Comparing male and female patients, it can be noticed that all entities are represented more often in male patients.

We analyzed 144,969 cases of head and neck cancer and selected 62,775 cases of OPC (ICD–10: C01, C09, and C10) for a detailed analysis. The consolidated standard of reporting trial (CONSORT) flow diagram, shown in **Figure 1**, illustrates the data extraction process from the TriNetX real–world database (database access on 7 December 2020).

In **Table 1**, the total number of cases sorted by different ICD–10 codes is displayed, separately broken down to male and female and relative frequency of each cancer entity within the comparison.

**Table 1:** Characteristics of the full cohort with frequencies of tumor entities and sex.

ICD–10	Region	Number of Cases	Frequency	Male	Female
			(in percent)		
C00	Lip	5,221	4	67	33
C01	Base of tongue	17,544	12	79	21
C02	Other and unspecified parts of tongue	26,345	18	68	32
C03	Gum	5,637	4	55	45
C04	Mouth floor	8,120	5	67	33
C05	Palate	5,763	4	59	41
C06	Mouth	22,460	16	63	37
C09	Tonsil	18,699	13	80	20
C10	Oropharynx	17,731	12	77	23
C11	Nasopharynx	8,388	6	64	36
C12	Sinus	3,109	2	82	18
C13	Hypopharynx	5,942	4	77	23
	All cases	144,969	100	70	30

The sum of cases amounts to 144,969 with a percentage distribution of 70% male and 30% female patients.

The prominence of malignant neoplasm of base of tongue (C01) is notable, with a frequency of 18% and a total of 26,345 patients, it is the most common carcinoma in this study, followed by mouth cancer (C06) with a frequency of 16%.

In terms of the distribution of men and women, it is noticeable that the percentage for each ICD–10 code of male patients is higher compared to the proportion of female patients.

C12, malignant neoplasm of pyriform sinus, has the highest proportion of male patients with 82%, with a total frequency of only 2%.

The largest distribution of female individuals can be seen in C03, malignant neoplasm of the gum, in which both sexes are almost evenly distributed (male patients, 55%, female patients, 45%).

The sex inequality in the 5–year survival rate with and without HPV–positive oropharyngeal carcinoma is illustrated in **Table 2**. It differentiates between male and female individuals with oropharyngeal cancers (C01, C09, and C10) tested either positive or negative for HPV. In the center column, the HR between male and female patients is applied.

**Table 2:** Survival rates in percent for oropharyngeal carcinomas, subdivided by diagnosis (ICD–10: C01, C09, and C10), sex (male, female) and HPV status (HPV±).

Oropharyngeal Carcinomas(ICD–10: C01, C09, and C10)								
	Male				Female			
	Survival rate (%) after propensity score matching			♂ vs.♀HPV+	Survival rate (%) after propensity score matching			
Entity of tumor	HPV−	Hazard Ratio (CI)	HPV+	Hazard Ratio (CI)	HPV+	Hazard Ratio (CI)	HPV−	Entity of Tumor
Tongue base	79.3	0.971 (0.62–1.521)	78.8	0.835 (0.457– 1.524)	83.7	0.851 (0.378–1.918)	81.3	Tongue base
Tonsil	87.8	0.8 (0.51–1.257)	83.8	0.578 (0.262–1.281)	90.3	2.157 (0.86–5.408)	75.5	Tonsil
Oropharynx	66.3	1.871 (1.284–2.724)	81.5	1.077 (0.602–1.926)	80.6	2.148 (1.136–4.062)	61.0	Oropharynx

Hazard ratios were calculated for HPV–positive and HPV–negative cases as well as for HPV–positive male and female cases (middle), confidence intervals (CIs) are given in brackets.

Comparing cancers with varying localization (C01, C09, and C10) within male patients, it can be found that the survival rates between patients tested HPV positive and HPV negative differs. Individuals with oropharynx carcinomas (C10) who tested HPV negative have a survival rate after propensity score matching of 66.3%, in turn, patients who tested HPV positive occurred to have a significantly higher survival rate of 81.5%. The HR of 1.077 after age/sex matching shows that, in this cancer entity, HPV infection is associated with a survival advantage.

For C09, neoplasms of the tonsils, it occurs that HPV–negative male patients have an 87.8% chance of surviving, which is 4% higher than male HPV positively tested C09 patients.

In the sub–cohorts of female patients, tonsil (C09) and oropharynx (C10) carcinomas in the HPV–positive cohorts have a 15%–20% higher survival rate in comparison with the HPV–negative female patients, with a HR larger than 2.

The disparities do not only appear within one sex, differences in survival rate and HR are also noticeable between both sexes. Malignant neoplasms of tongue base (C01) and tonsils (C09) have an almost 6%–7% higher survival rate for female patients with HPV. Only in malignant neoplasms of the oropharynx male and female survival rates did hardly differ from each other.

**Figure 2:**
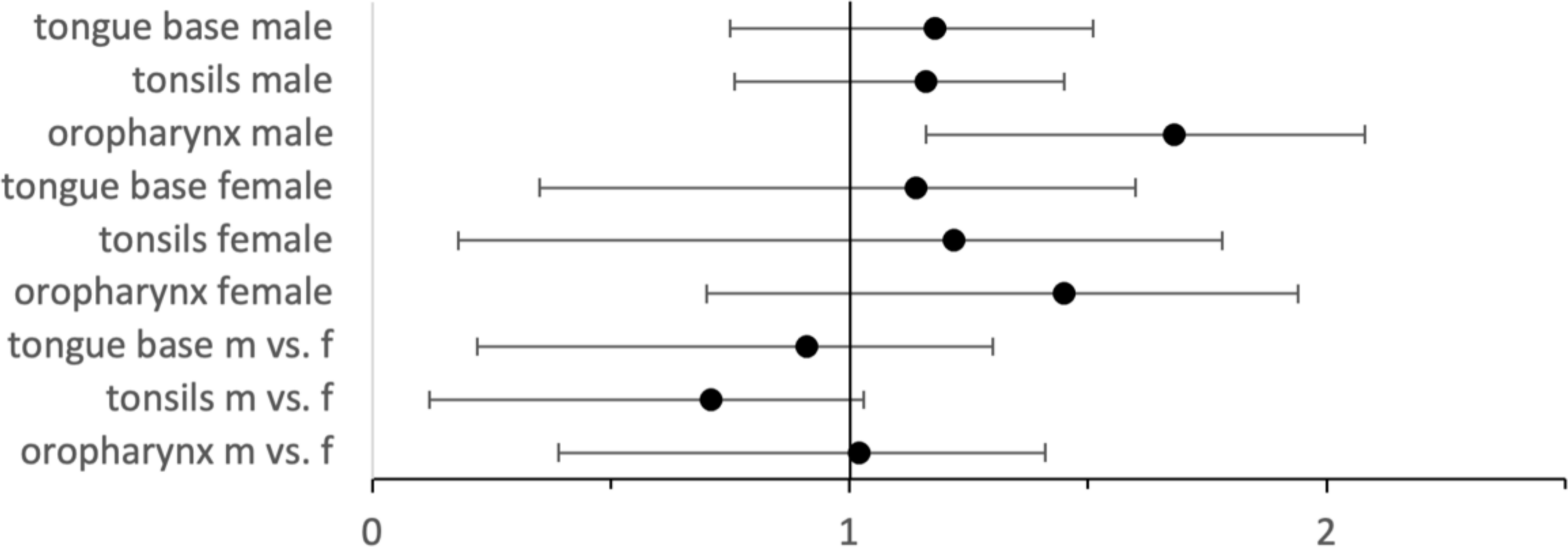
Hazard ratios with 95% CIs for HPV–positive vs. HPV–negative cases for male and female cases as well as for HPV–positive male vs. female cases.

A further analysis of all cohorts with 1:1 matching regarding alcohol and nicotine dependence (**Figure 2**) was performed, resulting in only slightly different results in HRs.

**Figure 3:**
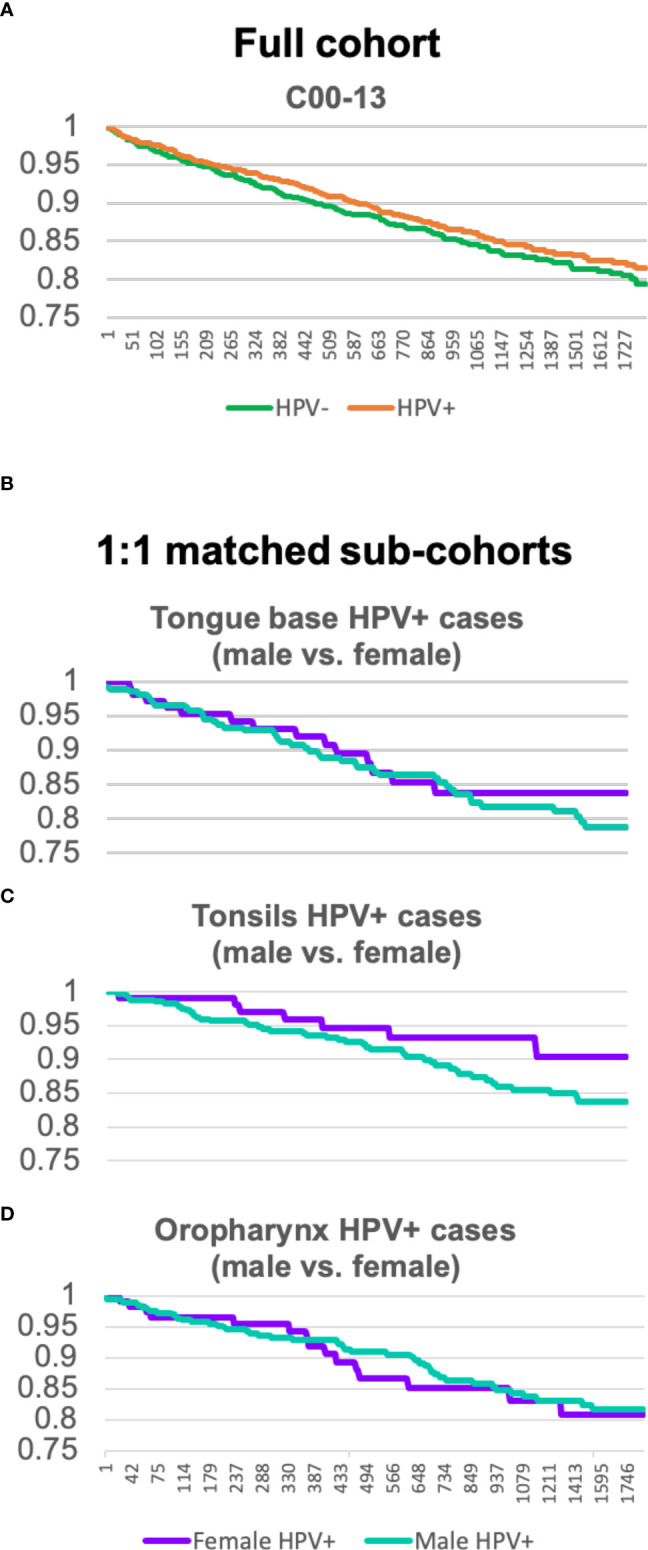
**(A)** Kaplan–Meier analysis comparing the full cohort of OPC patients tested HPV–positive and HPV negative. **(B–D)** Kaplan–Meier analysis of sex–matched sub–cohorts of tongue base, tonsils, and oropharynx carcinoma patients with HPV comparing male (green) and female (purple) individuals.

Looking at Kaplan–Meier analysis **Figure 3A**, which compares the survival rate of HPV–negative (green)– with HPV–positive (orange)–tested OPC patients], it can be seen that HPV–positive patients have a slightly higher probability of surviving within 5 years (from event occurrence to end of trial). HPV–positive cohorts seem to have an advantage.

For HPV–positive OPC (C01, C09, and C10), Kaplan–Meier analysis for **Figures 3B** **–D** was created separately and shows sex–matched sub–cohorts of tongue base, tonsils, and oropharynx carcinoma patients with HPV, respectively.

For tongue base neoplasms (C01), male (green) or female (purple) individuals display a similar trend up to 2.5 years. After this time, a divergency between the survival rate of female and male patients can be seen. Female patients are more likely to survive a neoplasm of tongue base than HPV–positive male patients.

Whereas the divergence for C01 firstly appears after 2.5 years, the survival rate of the sub–cohort of C09 of the two sexes departs after 114 days. Kaplan–Meier analysis C exhibits quite a significant disparity, the curve of female patients shows a slow decline and keeps a steady level of 0.9 after 1,211 days, whereas the male sub–cohort continuously decreases down to 0.83.

Kaplan–Meier analysis D, neoplasms of the oropharynx (C10), depicts a similar development between the survival rate of female and male patients.

In conclusion, HPV is a better prognostic marker for female tongue base and tonsil carcinoma patients than it is for HPV–positive male patients.

## Discussion

Traditional risk factor for the onset of head and neck squamous cell carcinomas (HNSCCs), which comprise of cancers of the oral cavity (OSCC) as well as OPC, larynx, and hypopharynx, is the use of nicotine and alcohol. Nevertheless, this study compared a number of factors influencing the survival (not as hazard factor) and found a lower influence of nicotine and alcohol dependence compared to HPV status. An exemplary list of confounders (from diagnoses, lab values, medications, and demographics) and their influence on survival is given in the **Supplementary Material** (**Additional File 14**), showing that the HPV± status has the strongest impact.

An increasing number of HNSCCs can be associated with infection with the human papillomavirus (HPV). In the United States alone, the incidence of HPV–positive OPC increased by about 225% between 1988 and 2004, as is considered to be responsible for up to 90% of all new OPCs nationwide (2, 19). In particular, for OPC, a significant correlation between HPV infection and a more favorable prognosis of the disease has been reported. Despite the increasing incidence of this entity, young and healthy patients, affected by HPV–positive OPC, are still an unfamiliar cohort, seeking medical advice for cancer symptoms, delaying the diagnosis and treatment (19). Infections with HPV are being classified as STD, accounting for the most common STD according to the WHO. However, as transmission occurs through sexual contact, most infections remain asymptomatic and are spontaneously cleared within the first 2 years. Highly oncogenic subtypes of HPV are described in a previous study (20). In particular, HPV–16 is known to be responsible for most HPV–related cancer cases, making it susceptible to vaccination (21). HPV–positive OPC patients have significantly better prognosis than HPV–negative OPC patients. Standardized HPV status assessment is now used for risk stratification on a routine basis. Ang et al. described a 3–year overall survival (OS) of 82.4% in HPV–positive disease and 57.1% in HPV–negative disease, respectively. In HPV–positive OPC patients without a history of smoking and no lymph node involvement, 3–year OS rates of 93% seem reasonable (22). This is also true for long–term follow–up as Posner and colleagues’ report on a 5–year OS of 82% for HPV–positive versus 35% for HPV–negative OPC patients (23).

As an additional parameter in OS analyses of HPV–positive HNSCC, several studies have identified the racial background of the patients. Not only the rates of HPV infections differ between black and white study populations, but also the survival rates are supposedly better in African Americans with HPV–positive OPC (24, 25). The influence of sex on OS in OPC has been published in a smaller study population by Fakhry et al. in 2017 (26). HPV–positive OPC women were shown to have a survival advantage in comparison with men. Although the incidence of HPV–positive OPC is lower among women than men, the vast majority of OPC in women are HPV positive (26). Our data, including a larger study population, show a general higher OS in HPV–positive OPC despite the specific anatomical location. This is congruent with reports from the literature. However, in sub–analysis of anatomical locations and sex, differences in OS between male and female patients could be observed. Our data show significantly better survival rates for women with cancers of the tongue base and the tonsils compared to men with HPV–positive cancers in the same regions. Although these findings support the data published by Fakhry et al. in a smaller study population, a reliable explanation is still missing. Female sex indeed seems to be an independent prognostic factor for OS in OPC. This has been subject to research in other tumor entities, leaving male subpopulations with worse survival rates than female ones (27). One explanation could be that women have less tobacco and alcohol exposure than men, still being a cofounder even in HPV–positive OPC. For non–squamous cell lung cancers, improved survival rates in female patients are explained by distinct phenotypes of disease by sex rather than smoking as a risk factor (28). Moreover, comorbidities and general death rates could also add up to this equation. This current study underlines the role of sexes as an independent risk factor in HPV–associated OPC providing a large sample size. Nevertheless, the retrospective study design and the large cohort cannot reveal differences in tobacco and alcohol abuse and comorbidities. The necessity of HPV testing is emphasized by the results of this study, identifying patients at risk for disease recurrence. Moreover, the biological sex should be established as an independent risk factor.

Because of the large cohorts, RWE studies tend to provide useful data to recognize trends, but their validation by prospective clinical trials as the gold standard is crucial to eliminating weaknesses in data like the unclear origin of HPV test samples.

## Conclusion

Sex disparities, differences between tumor entities, and HPV diagnoses result in varying survival rates and reveal the necessity of customized, patient–specific prognoses and therapies. Causes for these disparities demand further investigation and warrant a re–evaluation of therapy strategies to improve clinical practical guidelines and enable optimal treatment for patients with oropharyngeal cancer.

## Data availability statement

The datasets presented in this study can be found in online repositories. The names of the repository/repositories and accession number(s) can be found in the article/**Supplementary Material**.

## Ethics statement

TriNetX is compliant with the Health Insurance Portability and Accountability Act (HIPAA), the US federal law that protects the privacy and security of healthcare data. TriNetX is certified to the ISO 27001:2013 standard and maintains an Information Security Management System (ISMS) to ensure the protection of the healthcare data it has access to and to meet the requirements of the HIPAA Security Rule. Any data displayed on the TriNetX Platform in aggregate form, or any patient–level data provided in a dataset generated by the TriNetX Platform, only contain de–identified data as per the de–identification standard defined in Section §164.514(a) of the HIPAA Privacy Rule. The process by which the data is de–identified is attested to through a formal determination by a qualified expert as defined in Section §164.514(b)(1) of the HIPAA Privacy Rule. This formal determination by a qualified expert, refreshed in December 2020, supersedes the need for TriNetX’s previous waiver from the Western Institutional Review Board (IRB). The TriNetX network contains data provided by participating Healthcare Organizations (HCOs), each of which represents and warrants that it has all necessary rights, consents, approvals, and authority to provide the data to TriNetX under a Business Associate Agreement (BAA), so long as their name remains anonymous as a data source and their data are utilized for research purposes. The data shared through the TriNetX Platform are attenuated to ensure that they do not include sufficient information to facilitate the determination of which HCO contributed which specific information about a patient. This study was reviewed and approved by Ethikkommission der Charité - Universitätsmedizin Berlin, EA4_064_18. Written informed consent was obtained from all participants for their participation in this study.

## Author contributions

SHP analyzed and interpreted data, wrote the first version of the manuscript, and designed the figures. SN analyzed and interpreted data. SP conceived the study, performed the analysis, and wrote the manuscript. MH analyzed and interpreted data. SK analyzed and interpreted data and wrote the manuscript. All authors read and approved the final version of the manuscript.

## Funding

This work was funded by the German Research Foundation (DFG: PR 1562/1–1).

## Conflict of interest

The authors declare that the research was conducted in the absence of any commercial or financial relationships that could be construed as a potential conflict of interest.

## Publisher’s note

All claims expressed in this article are solely those of the authors and do not necessarily represent those of their affiliated organizations, or those of the publisher, the editors and the reviewers. Any product that may be evaluated in this article, or claim that may be made by its manufacturer, is not guaranteed or endorsed by the publisher.
